# Body Mass Index Effects Kruger’s Criteria in Infertile Men

**DOI:** 10.22074/ijfs.2018.4888

**Published:** 2017-10-14

**Authors:** Yaprak Engin-Ustun, Nafiye Yılmaz, Nilufer Akgun, Ayla Aktulay, Ahmet Deniz Tuzluoğlu, Batuhan Bakırarar

**Affiliations:** 1 Zekai Tahir Burak Education and Research Hospital, Reproductive Endocrinology Unit, Ankara, Turkey; 2 Zekai Tahir Burak Education and Research Hospital, Urology Unit, Ankara, Turkey; 3 Department of Biostatistics, Faculty of Medicine, Ankara University, Ankara, Turkey

**Keywords:** Body Mass Index, Male Infertility, Obesity

## Abstract

**Background:**

The aim of this study is to evaluate the relationship between sperm parameters and body mass index (BMI) in the male spouses with infertility complaints, who had reffered to our clinic.

**Materials and Methods:**

The male spouses from 159 couples reffering to our clinic because of infertility, during a six-month period, were included in the study. In this prospective case control study, the included men were catego- rized as non-obese (BMI<25 kg/m^2^), overweight (BMI 25-29 kg/m^2^) and obese (BMI ≥30 kg/m^2^) according to their BMIs. The assessed sperm parameters consisted of; sperm concentration, Kruger morphology, progressive motility level, and volume pH levels. The statistical significant level was set as less than 0.05 .

**Results:**

The assessed group consisted of 159 patients applying to our clinic with infertility symptoms. Fifty-three non-obese, 53 overweight and 53 obese men were eligible for the study. There was statistically significant differences in sperm volume (P<0.001), progressive motility (P<0.001), postwash sperm count (P<0.001) and Kruger (P<0.001) morphology among the patient groups grouping according to the BMI levels.

**Conclusion:**

In this study, increased BMI was associated with decreased semen quality, affecting volume, concentra- tion, and motility. further studies with a wider range of prospective cases need to be conducted in order to investigate the effects on male fertility in more detail.

## Introduction

The definition of obesity given by the World Health Organization (WHO) is; a body mass index (BMI) of over 30 kg/m^2^ (1). The epidemic global obesity displays a parallel relation with the decrease in semen quality. The connection between the sperm parameters and obesity is debatable. Obesity reduced semen quality and the sperm mitochondrial activity. Also itincreases sperm DNA damage and miscarriage, induces seminal oxidative stress, impairs blastocyst development, or reduces pregnancy outcome following assisted reproduction (2). Furthermore; there are other outcomes such as impaired erectile function, and other physical problems, such as increased scrotal temperatures and sleep apnea (3, 4). The male reproductive function can be significantly affected by the hormonal changes related to obesity. A reduction in the binding capacity of sex hormone-binding globulin with increased serum oestradiol reduced luteinizing hormone (LH) pulse amplitude. Decreased levels of serum gonadotropin and testosterone may be among these changes (5). Although there are conflicting reports in terms of obesity and sperm parameters; insulin-suppressed sex hormone binding globulin (SHBG) in obese men increases the androgen availability required by the adipose aromatase for the production of estrogen which may result in a reduced gonadotropin secretion (2).

In the subfertile couples, the increased abdominal adiposity in men is related to decreased concentration, motility, and sperm count (6) but it is also associated in some studies (7,8) not all (9,10) with an increased incidence of asthenozoospermia and oligozoospermia. In this present prospective study, the aim was to look into various semen parameters (sperm concentration, semen volume, morphology) in infertile obese-men and also to evaluate their association with BMI. 

## Materials and Methods

This study was conducted at the Zekai Tahir Burak Women’s Health Education and Research Hospital, Ankara from February 2016 to June 2016. The study protocol was in conformity with the principles of the Declaration of Helsinki, and approval of the institutional review board obtained. Each patient had his informed consent. In this prospective case-control study, the sample group consisted of 159 infertile Turkish men from central Anatolia between the ages of 22-54 years. Initially, all men were admitted to our infertility outpatient clinics with their spouses. Medical and infertility history of each patient was recorded. Also patients were excluded if any chronic diseases (chronic liver and kidney problem, asthma, epilepsy, steroid users, chronic inflammatory bowel disease, history of thromboembolism and cerebrovascular event, hypotroidea) were evident. They measured each patients’ weight and height with a professional, calibrated device. BMI was calculated by dividing the weight to height squared. Patients were stratified according to their BMI into three groups. The evaluation process included a total of 159 patients with infertility symptoms. Fifty-three non-obese (BMI<25 kg/m^2^), 53 overweight (25-29), 53 (BMI≥30 kg/m^2^) obese men were eligible for the study.

They asked the patients to refrain from sexual activity for a period of 3 days. They collected semen samples from the patients by masturbation in a private room nearby the laboratory. The collected semen specimens were assessed by using a computerized semen analyzer for conventional semen parameters including sperm motility and sperm concentration (pre-washed for half an hour at room temperature) after liquefaction. Standard swim-up method with a sperm preparation media (Ferticult Flushing medium TM, FertiProNV, Beernem, Belgium) was used to process the rest of the semen. A computer-assisted semen analyzer was used for the post-wash analyses. In relation to the quality control program; the same andrology laboratory technician performed the sperm analyses. 

The World Health Organization guidelines (11) were used to assess the sperm analyses. For each man sperm count, pH, semen volume, percentages of motility, sperm concentration, and normal sperm morphology were recorded. 

### Statistical analysis

All statistical analyses were performed by using SPSS for Windows 11.5 software program (SPSS Inc., Chicago, IL). Except descriptive statistics, one-way ANOVA and Kruskal Wallis were used to approximate statistical disparity on the collected data between BMI groups. A statistically significant difference was found between the group while evaluating the quantitative data of more than two groups. Also Post Hoc analyses were performed with Tukey HSD Test for the normally distributed data and paired Mann Whitney-U Tests conducted for abnormally distributed data and finally Bonferroni correction was applied. The statistical significance level was set as less than 0.05. The significance limit in the Bonferroni correction was determined to be 0.05/k with the evaluation number of the paired Mann Whitney-U being “k”. The statistical significance level was set as less than 0.05. 

## Results

The evaluated group consisted of 159 patients applying to our clinic with infertility symptoms. A total of 159 patients were grouped as non-obese (BMI<25 kg/m^2^) (53 men), overweight (BMI 25-29 kg/m^2^) (53 men) and obese (BMI ≥30 kg/m^2^) (53 men). The demographic and clinical characteristics of the patients has been shown (Table 1).

**Table 1:** Comparison of demographic and clinical characteristics of the patients


Variable	Obese group BMI≥30 kg/m^2^n=53		Overweight group BMI 25-29 kg/m^2^ n=53		Non obese group BMI<25 kg/m^2^ n=53		P value
	Mean ± SD	Median(Minimum-Maximum)	Mean ± SD	Median(Minimum-Maximum)	Mean ± SD	Median(Minimum-Maximum)	

Age (Y)	33.32 ± 6.64	32.00(23.00-54.00)	32.93 ± 4.92	32.00(22.00-45.00)	32.21 ± 5.82	32.00(24.00-52.00)	0.610
Sperm volume (cc)	1.97 ± 1.61	2.00(0.00-6.00)	3.36 ± 1.43	3.00(1.00-8.00)	2.94 ± 1.20	3.00(1.00-6.00)	<0.001^*^
pH	6.30 ± 3.27	8.00(0.00-8.00)	7.83 ± 0.24	8.00(7.50-8.00)	7.88 ± 0.43	8.00(6.00-8.00)	0.033^*^
Concentration (m/mL)	23.07 ± 30.38	12.00(0.00-150.00)	44.12 ± 47.13	25.00(0.00-170.00)	45.39 ± 45.36	33.00(0.00-180.00)	0.014^*^
Progressive motility (%)	21.49 ± 21.44	11.00(0.00-60.00)	40.60 ± 11.21	39.00(19.00-75.00)	41.02 ± 11.89	44.00(4.00-57.00)	<0.001^*^
Postwash sperm count (m/mL)	8.40 ± 11.05	5.00(0.00-43.00)	26.10 ± 19.62	21.00(2.00-65.00)	29.66 ± 19.39	22.00(2.00-65.00)	<0.001^*^
Postwash progressively motility (%)	38.63 ± 42.66	19.00(0.00-100.00)	27.75 ± 13.91	27.00(10.00-70.00)	29.60 ± 12.46	33.00(2.00-48.00)	0.446^*^
Morphology (%)	3.15 ± 2.93	3.00(0.00-10.00)	5.90 ± 2.09	6.00(3.00-11.00)	6.09 ± 2.28	7.00(2.00-13.00)	<0.001^*^
Concentration<15 m/cc n (%)	1.00 ± 0.00	1.00(1.00-1.00)	1.00 ± 0.00	1.00(1.00-1.00)	1.00 ± 0.00	1.00(1.00-1.00)	1.000^*^
Azoospermian (%)	1.00 ± 0.00	1.00(1.00-1.00)	1.00 ± 0.00	1.00(1.00-1.00)	1.00 ± 0.00	1.00(1.00-1.00)	1.000^*^


*; Kruskal Wallis Test analysis results and BMI; Body mass index.

Statistically significant differences were found in sperm concentration among the three groups (P=0.014). The statistically significant difference was because of the difference between the obese and non-obese groups (P=0.019). 

There were also statistically significant differences in sperm volume (P<0.001) among three groups. The statistically significant difference was because of the difference observed between the obese and non-obese groups (P=0.010) and also the obese and overweight groups (P<0.001). Also There were statistically significant differences in pH among three groups (P=0.03). The statistically significant difference was because of the difference observed between the obese and non-obese groups (P=0.010) and also the obese and overweight groups (P=0.007). There were also statistically significant differences in progressive motility among three groups (P<0.001). The statistically significant difference was due to the difference between the obese and non-obese groups (P<0.001) and the obese and overweight groups (P=0.001). There were also statistically significant differences in postwash sperm count among three groups (P<0.001). The statistically significant difference was due to the difference observed between the obese and non-obese groups (P=0.008) and the obese and overweight groups (P=0.013). There were also statistically significant differences in Kruger morphology among three groups (P<0.001). The statistically significant difference was due to the difference observed between the obese and non-obese groups (P<0.001) and the obese and overweight groups (P<0.001). 

## Discussion

The principal finding of this study is that; in terms of semen parameters there are statistically significant differences among the non-obese, overweight and the obese infertile patients. The effect of obesity on sperm quality is still under discussion. Nearly 40% of all infertility cases have a male origin (12). The reasons for male infertility may be numerous. Infertility may be induced in males by a number of reasons; the presence of antibodies against sperm, sperm production disorders, hormonal disorders, blockage in the sperm ducts, testicular trauma, varicocele, anatomical problems, certain medications and infections can be named among them. Hence, for eliminating factors that could play a role in changing the semen parameters alongside BMI, we excluded factors such as smoking, varicocele, hormonal changes, and other fertility-affecting factors in our study. Based on the recent reports by the WHO, obesity is a major concern; as in 2008; globally more than 1.4 billion were overweight and 500 million were obese (13). Nevertheless, theeffect of obesity on sperm parameters is conflicting. Although the semen parameters established by the WHO were used in various studies, the results obtained on the impact of obesity on sperm parameters were dissimilar. 

The sperm concentration of infertile men in this study displayed a statistically meaningful relation with BMI. According to the recent WHO surveillance study; conducted on male partners of pregnant women; determined that the total sperm count of the obese-men were significantly lower than the non-obese men (mean 231×10^6^ vs. 324×10^6^, respectively), even though other sperm parameters did not reveal any signs of being affected (14). Hofny et al. (15) demonstrated decreased sperm count in obese normozoospermic men compared with nonobese fertile subjects. Bounartzi et al. (16) showed that overweight and obese men had reduced sperm volume and the BMI was correlated with the percentage of degenerated spermatozoa and increased sperm DNA fragmentation. Belloc et al. (17) revealed that increased BMI was associated with decreased semen quality which affects volume, concentration, and motility.

Our results are contradictory with those published by the analysis of a database of 2139 patients, which displayed no significant change in obese men. The effect of it on sperm count may be negligible (mean total sperm count for BMI 20-25 kg/m^2^: 231×10^6^; BMI.30: 265×10^6^) (5). According to Martini et al. (18), no relationship between BMI and sperm concentration was noted. Similar findings were mentioned in other studies (19, 20). Still, conflicting with these studies; a study, including healthy volunteers showed that overweight men, in comparison to men with normal BMI, had significantly lower total sperm count and sperm concentration (21). Our results display a significant association between the semen volume and BMI. Wang et al. (22) revealed lower sperm quality (total sperm count, sperm concentration, motile sperm, relative amounts of type A motility, and progressive motility sperm [A + B]) in overweight and obese participants than in those with normal body mass index. In a more recent study by Rosenblatt et al. (23) it was shown that among the obese men the sexual quality of life was diminished, and increased BMI correlated with reduced sexual activity. A large clinical study conducted by Beloc et al. (24) on >10,000 samples; displayed that there was a distinct link between obesity and sperm production (total sperm count, volume and, concentration), whereas no other statistically significant relationship was found in terms of other semen parameters, especially the sperm morphology.

Evaluation of the sperm morphology may be disputable since it continues to be affected by influences of the subjectivity of the observer and because of the lack of objective measurement standards. A statistically significant association between the sperm morphology and BMI was observed by us. A study with a small sample size demonstrated that weight loss led to improvement in semen quality including sperm count and normal sperm morphology (25). The results of a study declared that there was a significant positive correlation between abnormal sperm morphology and BMI (15). In order to have a better idea about the effects of excess weight or obesity on reproduction; men should be included in the studies notonly for the BMI butalso for the factors related to excess weight which could also affect fertility; such as fat distribution, lifestyle habits, and associated pathologies. As an example, Hammiche et al. (26), noted that not only the BMI, but also another factor such as waist circumference of 102 cm, which is a measure of central adiposity, was inversely associated with total motile sperm count and sperm concentration. Håkonsen et al. (27), came to the conclusion that; weight loss could improve the semen quality in a study with a relatively low sample size. In addition Anifandis et al. (28) the sperm DNA integrity, as a potential clinical marker of semen quality SDF only correlated with sperm characteristics. 

On the other hand, while BMI of men affects fecundity, it is ambiguous that BMI has really an empact on sperm parameters. A retrospective study embracing in 301 subjects was carried out by Anifandis et al. (29) who unveils that there is an affiliation between male BMI and embryo quality; while male BMI does not correspond to sperm parameters which is no effect on the *in vitro* fertilization (IVF) outcome as well. Besides, Thomsen et al. (30) conducted on 612 infertile couples submitted to ART at a Danish fertility center. Semen parameters (sperm concentration, total sperm count, seminal volume and motility) were statistically insignificant influenced by male BMI. Furthermore, fertilization rate, number of good quality embryo, implantation and pregnancy as the outcomes of ART were not depended upon by the escalation of male BMI. One prime limitation of our study was that due to the low number of patients, it could not be a populationbased study. Also there were no records of whether the same patients had multiple sperm analyses during the study period and how they were conducted. There was also another limitation due to the heterogeneity of the populations (both general population and infertile couples) under focus. On the other hand, one of the important sperm characteristics, sperm DNA fragmentation (SDF) data were not included in our study due to insufficient funds.

## Conclusion

The effect of obesity on the conventional semen parameters in infertile men was evaluated in the study. In this study, we established statistically significant differences between the BMI and sperm concentration, motility, semen volume, and normal sperm morphology. Large prospective randomized clinical studies, including men with no infertility problems should be conducted in order to support these findings and investigate the effects on male fertility in more detail. Further studies at the level of advanced semen parameters such as the sperm DNA integrity are needed. 
